# Fitness difference between two synonymous mutations of *Phytophthora infestans* *ATP6* gene

**DOI:** 10.1186/s12862-024-02223-4

**Published:** 2024-03-18

**Authors:** Oswald Nkurikiyimfura, Abdul Waheed, Hanmei Fang, Xiaoxian Yuan, Lixia Chen, Yan-Ping Wang, Guodong Lu, Jiasui Zhan, Lina Yang

**Affiliations:** 1 Institute of Plant Virology, Ministry of Education, Fujian Agriculture and Forestry University, Fuzhou, Fujian 350002 China; 2 Fujian Key Laboratory on Conservation and Sustainable Utilization of Marine Biodiversity, Fuzhou Institute of Oceanography, Minjiang University, Fuzhou, 350108 China; 3 College of Resources and Environment, Fujian Agriculture and Forestry University, Fuzhou, Fujian, 350002 China; 4 College of Chemistry and Life Sciences, Sichuan Provincial Key Laboratory for Development and Utilization of Characteristic Horticultural Biological Resources, Chengdu Normal University, Chengdu, Sichuan 611130 China; 5 Department of Plant Pathology, Fujian Agriculture and Forestry University, Fuzhou, Fujian 350002 China; 6 Department of Forest Mycology and Plant Pathology, Swedish University of Agricultural Sciences, Uppsala, 75007 Sweden

**Keywords:** Disease management, Oomycete, Population genetics, Fitness of species, Neutral theory, Thermal-mediated adaptation, Plant pathogen

## Abstract

**Background:**

Sequence variation produced by mutation provides the ultimate source of natural selection for species adaptation. Unlike nonsynonymous mutation, synonymous mutations are generally considered to be selectively neutral but accumulating evidence suggests they also contribute to species adaptation by regulating the flow of genetic information and the development of functional traits. In this study, we analysed sequence characteristics of *ATP6*, a housekeeping gene from 139 *Phytophthora infestans* isolates, and compared the fitness components including metabolic rate, temperature sensitivity, aggressiveness, and fungicide tolerance among synonymous mutations.

**Results:**

We found that the housekeeping gene exhibited low genetic variation and was represented by two major synonymous mutants at similar frequency (0.496 and 0.468, respectively). The two synonymous mutants were generated by a single nucleotide substitution but differed significantly in fitness as well as temperature-mediated spatial distribution and expression. The synonymous mutant ending in AT was more common in cold regions and was more expressed at lower experimental temperature than the synonymous mutant ending in GC and vice versa.

**Conclusion:**

Our results are consistent with the argument that synonymous mutations can modulate the adaptive evolution of species including pathogens and have important implications for sustainable disease management, especially under climate change.

**Supplementary Information:**

The online version contains supplementary material available at 10.1186/s12862-024-02223-4.

## Background

Understanding the genetic mechanisms underlying specific adaptations has long been a central theme in evolutionary research. In plant pathology, this knowledge has important implications for sustainable disease management and food production. According to the central dogma of molecular biology, protein functions are determined by amino acid sequences, leading to adaptive polymorphism and local adaptation [1, 2]. Synonymous mutations, i.e., change in the DNA sequence does not affect the amino acid sequence in the protein [3], should not have effects on the fitness of organisms and are considered selectively neutral. However, recent findings from functional analyses, comparative genomics, and experimental evolution disagree this hypothesis.

Synonymous mutations usually occur in the third position of a codon, resulting in several codons for each amino acid [4]. Neutral theory expects all codons encoding the same amino acid are used equally but codon usage bias has been widely documented in nearly all species ranging from viruses [5, 6], bacteria [7, 8], fungi [9], plants [10, 11], insects [12] and humans [13]. In addition to unequal mutations [14] and mismatched tRNA availability [15], this codon usage bias among synonymous mutations could be partially associated with their functional differentiation. Indeed, in the molecular context, synonymous mutations are increasingly recognized as a mechanism that regulates information flow of genetic codes at multiple levels [16] through affecting mRNA stability, protein expression and enzymatic activity and thus are not evolutionarily neutral. For example, synonymous mutation can affect mRNA structure, and the formation of promoters, resulting in changes in translational efficiency and protein folding [17]. In African green monkey, synonymous mutations substantially influenced the folding process of a nascent protein and modified the substrate specificity of the enzymes [18]. In pathology, synonymous mutations frequently contribute to cancers [19] and mental diseases [20] of human and adaptation of *Methylobacterium extorquens* to ecosystems [21]. Therefore, synonymous mutations could affect species fitness in a variety of ways; thereby reshape the adaptive landscape of species [21, 22].

Most studies in synonymous mutations have focused on the issues associated with codon bias and their applications such as for molecular clock and phylogenic analyses [23]. Knowledge about the adaptive role of synonymous mutations is still fragmented, but can provide new insights into evolutionary processes and mechanisms that contribute to human health and ecological function [24]. To reach the overarching aim, we analysed the *ATP6* gene in 139 *Phytophthora infestans* isolates sampled from various ecosystems. *P. infestans*, an oomycete plant pathogen, is a model species for studying evolutionary biology. Given its ecological and agricultural importance [25, 26], the biology of the species has been well characterized [27, 28]. The pathogen grows best at 17 ∼ 20 °C [29] and could cause nearly 100% mortality of susceptible potato hosts within a week under conducive thermal (16–22 °C) and humidity (> 95%) conditions [30]. Being a deep and unique lineage of eukaryotic pathogens, *P. infestans* has now entered a post-genomic era. A multitude of genomes are available and have been analysed [31, 32], providing fundamental concepts of genomic structure to study the evolutionary role of synonymous mutations. Possibly due to its large genome size enriched with many transposable elements [33] and other biological and epidemiologic characteristics such as the ability of long-distance dispersal for gene flow and a mixing model of reproduction [34], the pathogen can evolve quickly in responding to ecological fluctuations [26, 35], enabling us to deduct genomic and phenotypic changes with a resettable time scale.

Housekeeping genes are unique for studying the adaptive role of synonymous mutations. Due to their conserved evolution, housekeeping genes usually exhibit reduced genetic variation mainly arising from synonymous mutations. *ATP6* is a housekeeping gene that encodes a protein essential for respiration and bioenergetics to secure normal cellular activities [36]. The protein is a subunit of ATP synthase responsible for F_O_-proton transmission in the oxidative phosphorylation chain that catalyses ATP production from ADP [37]. The energy for ATP formation is supplied by a rotational mechanism of the membrane-bound F_O_ moiety driven by the electrochemical gradient across the membrane through channels at the interface of the peripheral stator and the c-ring [38]. Environmental stress frequently leads to a decrease in net energy balance due to reduced energy absorption and/or conservation in the form of ATP and other high-energy phosphates (supply-side effects), increased basal metabolic demand (demand-side effects), or a combination of the two [39, 40]. The ensuing energy shortage can have a negative impact on organism survival and performance, as well as the long-term persistence of its populations in a stressed environment. However, species can genetically and physiologically respond to environmental stress, such as thermal conditions, through changes in the expression and sequence of associated genes and mitochondrial numbers [41, 42].

The specific objectives of this study were to: (i) investigate population genetic structure of *ATP6* gene in the late blight pathogen *P. infestans*; (ii) determine the types of sequence variation in *ATP6* gene; (iii) infer the effect of ecological factors on the population genetic structure of *P. infestans ATP6* gene; and (iv) infer the contribution of synonymous mutation to the ecological adaptation of *ATP6* gene in *P. infestans*. Understanding these questions would provide us fresh insights into the evolutionary roles of synonymous mutations with important implication for mitigating disease impacts on ecosystems and human society under future climate conditions. We found a single synonymous mutation in *ATP6* gene contributed remarkably to intrinsic growth, stress tolerance and virulence of *P. infestans* and its adaptation to chemical and temperature stresses.

## Materials and methods

In the study, the *ATP6* gene in the 139 genetically distinct *P. infestans* isolates were sequenced, and its spatial distribution was associated with the annual mean air temperature and geographic parameters of the source population. Fitness components, including aggressiveness, metabolic rates and tolerances to a fungicide (azoxystrobin) which is chosen due to its widespread usage, specific mode of action and documented resistance issues, cold, and hot of the isolates were determined and compared between the groups of isolates with synonymous mutations. Aggressiveness was measured by the amount of disease a pathogen isolate causing on a potato variety and azoxystrobin tolerance was measured by calculating the relative growth rate (RGR) of the pathogen in the presence and the absence of the fungicide. Metabolic rate of the pathogen was represented by the growth rate of the pathogen estimated at 19 °C while temperature sensitivity was estimated by calculating the relative growth rate of colonies at 13 or 25 °C to that at 19 °C.

### Pathogen collections

During the 2010 and 2011 growing seasons, 100–200 infected potato leaves with symptoms of *P. infestans* were collected from each of seven fields covering various climatic zones and representing important potato growing areas in China. The diseased leaves, taken at random from potato plants spaced 1–2 m apart, were transported to the laboratory in separate sandwich bags for isolation of *P. infestans*. A thorough cleaning process that involved rinsing with tap water and sterilizing with distilled water was performed to remove mud from the diseased leaves, which were then placed abaxial side up on 1.0% water agar for 20–30 h to sporulate. A single piece of mycelium was taken aseptically from the edge of the sporulating lesion using a sterilized needle, transferred to a rye B agar plate amended with antibiotics (100 µg/mL ampicillin and 10 µg/mL rifampicin), and incubated at 19 °C in the dark for seven days to develop a colony. Purification was performed by transfers of a single sporangia to a fresh rye B plate. A total of 716 isolates secured from the collections were first genotyped by eight SSR loci, 11 differential varieties, PCR amplification of mitochondria and mating type [43, 44] and then stored long-term at 13 °C until further use. *P*. *infestans* could lose viability, such as pathogenicity after long-term storage on media [45]. These biological features were restored, when necessary, by infecting a susceptible potato cultivar. Details of pathogen collection, isolation, and restoration are described in previous publications [29, 46].

### PCR and *ATP6* gene sequencing

Pathogen isolates with different SSR alleles, virulent profiles (i.e., race structure), and mating types were considered to be different genotypes. As a result, 139 *P. infestans* genotypes detected in the collections. This pattern of genetic variation is similar to previous studies in China and other parts of the world, showing that *P. infestans* populations were dominated by a small number of clones accompanied by many novel genotypes at very low frequencies. For example, 68 and 80 distinct genotypes were detected in 229 and 96 isolates from China [47, 48] while 89 genotypes were detected in 237 isolates from Poland [49], respectively. Only one isolate with the same genotype was selected for the sequence analysis of the *ATP6* gene, resulting 139 isolates in total. These isolates, with 15–30 representatives from each of the seven populations (Table 1), were retrieved from the long-term storage and cultured on rye B agar at 19°C under dark conditions. Mycelia were harvested after two weeks of culture and freeze-dried. Genomic DNAs of the isolates were extracted from the lyophilized mycelia (∼ 100 mg/isolate) using gDNA Kit (Pomega Biotch. Co. Ltd., Beijing). PCR amplification of the *ATP6* gene was performed using *ATP6*-specific primers (PiATP6 For: 5´-GAAGCTGCTGCATGGTATTGG-3’, and PiATP6 Rev: 5´-GCGACCTATAGCGTCACAAGC-3’). The PCR amplifications were conducted in a 25µL reaction buffer using Gene Cycler TM (Bio-Rad), and the products were loaded onto gel electrophoresis (1%) for purification and single-direction sequencing as suggested by the manufacturer (QIA quick® Gel Extraction Kit). The sequencing was performed by GenScript Biological Technology Co., Ltd. (GenScript, Nanjing, China) using an ABI3730 automated DNA sequencer (Applied Biosystems, USA) and the full sequences were deposited in NCBI with accession numbers KX999558-KX999649 and KX999653-KX999699. The detailed protocols for DNA preparation and sequencing were described in previous publications [50, 51].

**Table 1:** Sample size, geographic origin, and sequence variation of the *ATP6* gene in the *P. infestans* populations collected from seven locations in China

Pop	Sample Size	Temp (°C)	Longitude	Latitude	Alt (m)	S	H	HD	Π
Fuzhou	21	20.5	119°17’	26º05’	10	1	2	0.381	0.00053
Xiapu	30	20.3	119°59’	26º54’	31	9	5	0.308	0.00092
Gansu	20	11.7	105°43’	34º35’	2089	4	4	0.432	0.00088
Guizhou	16	14.7	105°56’	26º16’	1330	1	2	0.525	0.00073
Ningxia	15	7	106°14’	36º01’	1778	1	2	0.133	0.00019
Guangxi	22	22.6	108°22’	22º50’	78	1	2	0.519	0.00072
Yunnan	15	15.6	102°43’	25º03’	2677	1	2	0.133	0.00019
Combined	139					12	7	0.539	0.00092

Sequence variation is measured by the number of polymorphic sites (S), number of haplotypes (H), haplotype diversity (HD), and nucleotide diversity (Π)

### Metabolic rate, temperature sensitivity, fungicide tolerance and aggressiveness assays

The 139 genetically distinct isolates were tested for metabolic rate, temperature sensitivity, azoxystrobin tolerance and aggressiveness according to a common garden design [52]. Metabolic rate, temperature sensitivity and fungicide tolerance were measured in vitro by quantifying pathogen growth on plates. For these measurements, the experiments were initiated by taking mycelia plugs (ϕ = 0.3 cm) from revived isolates and inoculated on rye B media either supplemented with azoxystrobin (0.01, 0.05, 0.10, 0.13, and 0.15 µg/mL) or without the supplementation of the fungicide in Petri dishes (ϕ = 9.0 cm). The inoculated plates were placed either on 13 °C (nearly lower boundary temperature), 19 °C (nearly optimum temperature) and 25 °C (nearly upper boundary temperature) of the pathogen growth [29] in growth chambers and were laid out in a completely randomized design using three replicates as recommended by previous studies [52]. Inoculated plates were kept in the dark at 19 °C and resultant colonies were photographed at the 3rd, 5th, 7th, 8th and 9th day after inoculation. Colony sizes were measured with the image analysis software Assess. The growth rate of each isolate was estimated using a logistic model [53] based on the sizes of individual colonies quantified at each time point over the inoculation period. The initial colony size at the point of inoculation (day one) was set as 0.2 cm^2^ (πr^2^ = 3.14 × 0.25^2^), and the capacity of colony sizes was set to 63.59 cm^2^ (πr^2^ = 3.14 × 4.50^2^). Metabolic rate was represented by the growth rate of the isolates at 19 °C. Temperature sensitivity was measured by calculating the growth rate of the isolates at 13 or 25 °C relative to that at 19 °C while azoxystrobin tolerance was measured by calculating the growth rate of the isolates in the presence of the fungicide relative to the absence of the fungicide.

Aggressiveness of the isolates was measured as the area under the disease progress curve (AUDPC; [54] according to the following formula [55].

$$ \text{AUDPC=}{\sum }_{\text{i=1}}^{\text{n}}\left(\frac{{\text{X}}_{\text{i+1}}\text{+}{\text{X}}_{\text{i}}}{\text{2}}\right)\left[{\text{t}}_{\text{i+1}}\text{-}{\text{t}}_{\text{i}}\right]$$

where *x*_*i+1*_ and *x*_*i*_ are disease severity at time *t*_*i+1*_ and *t*_*i*_, and *n* is the total number of observations, respectively. Disease severity was measured based on the size of lesions that appeared between Day 2 and 5 after inoculation on Favorita, a potato cultivar which is universally susceptible to *P. infestans*, using a detached leaflet approach [56]. To measure the severity of the disease, fully expanded leaves cut from Favorita grown for 8 weeks in the field were placed on 1% water agar in a Petri dish and then inoculated on the back of the leaves with mycelium plugs from the 139 isolates (φ = 0.5 cm) with three replicates. Petri dishes with detached leaves were arranged in a randomized complete block design and maintained for 16 h at 19 °C in an incubator with daily supplemental light. Leaves and lesion areas were photographed between the second and fifth days after inoculation and quantified electronically using Assess image analysis software [57]. Detailed protocols for experimental measurements of metabolic rate, temperature sensitivity, azoxystrobin tolerance and aggressiveness can be found in previous publications [25, 29, 55].

### Quantitative real-time PCR assays

A total of ten *P. infestans* isolates with five from each of two synonymous *ATP6* haplotypes (i.e., Hap_1 and Hap_2) were selected and grown under 13 and 25 °C for eight days. RNAs were extracted using a Eastep^®^ Super Total RNA Extraction Kit following the manufacturer’s instructions (Promega, Beijing, China). Their concentrations and purities (OD_260_/OD_280_ ratio > 1.95) were determined with a NanoDrop OneMicrovolume UV-Vis Spectrophotometer (NanoDrop Technologies, Wilmington, DE, USA) and cDNA was synthesized using PrimeScript™ RT reagent Kit with gDNA Eraser (Takara, Beijing, China), following the manufacturer’s instructions. qRT-PCR was performed on a CFX96 Real-Time System (BioRad), using Hieff^®^ qPCR SYBR Green Master Mix (Yeasen Biotech Co., Ltd., Shanghai, China) with specific *ATP6* QRT-PCR primers (For: 5´- TTGTATTTACTAACGCTTCT-3´ and Rev: 5´-ATATTTACCCGCTTTACC-3´). And the Actin A gene (For: 5´- CATCAAGGAGAAGCTGACGTACA-3´ and Rev: 5´-GACGACTCGGCGGCAG-3´) was used as the internal control. The expression of *ATP6* gene relative to the Actin A housekeeping gene was quantified using the 2 − ΔΔCT method [58].

### Meteorological data collection

Meteorological data for each collection site were downloaded from Weather Network (http://www.tianqi.com/). Annual mean temperature at collection sites was estimated using the air temperature across 10 years as described previously [29]. Geographic parameters of pathogen collection sites including altitude, latitude, and longitude were measured using a mobile compass.

### Statistical analysis

Before analysis, all *ATP6* nucleotide sequences were visually assessed to remove potential artifacts [59]. MUSCLE embedded in the software MEGA5 was used to perform the codon-based algorithm alignment. Nucleotide haplotypes were reconstructed with the PHASRE algorithm implemented in DnaSP 5.10 [60] and population diversity parameters including variation sites (S), the number of haplotypes (H), haplotype diversity (HD) and nucleotide diversity (Π) were estimated (Table 1). SSR, virulence and mating type data of the isolates were taken from previous publications [29, 61, 62]. Genetic differentiation in pathogen was participated hierarchically into among populations, among synonymous mutations and within synonymous mutations using the SSR data and difference in the population differentiation among and within the synonymous mutation were evaluated as described previously [63]. A dendrogram was depicted using the pairwise distances matrix [64] based UPGMA [65] and a principal component analysis (PCA) were performed using OriginPro, Version 2022 (OriginLab Corporation, Northampton, MA, USA).

A chi-square test for haplotype homogeneity among different populations was conducted by SPSS 19.0. A median-joining (MJ) network illustrating genealogical relationships among haplotypes was generated using PopART 1.7 [66]. A circle represented each haplotype and the proportion of sequences with a particular haplotype was indicated by circle size. Steps of nucleotide substitution among the haplotypes were indicated by number of black ticks.

Analysis of variance (ANOVA) of metabolic rate, temperature sensitivity, azoxystrobin tolerance, aggressiveness and expression level was performed using a general linear model implemented in SAS 9.4 (SAS Institute). Least significant differences (LSD) [67] were used to compare metabolic rate, temperature sensitivity, azoxystrobin tolerance, aggressiveness and expression level among *ATP6* haplotypes. In the ANOVA and LSD analysis, the five rare haplotypes were grouped together. Associations of haplotype frequency with meteorological and geographic landscape data in the seven populations were evaluated by simple linear correlation [68].

## Results

### Sequence characteristics of *Phytophthora infestans ATP6* gene

**Fig. 1:**
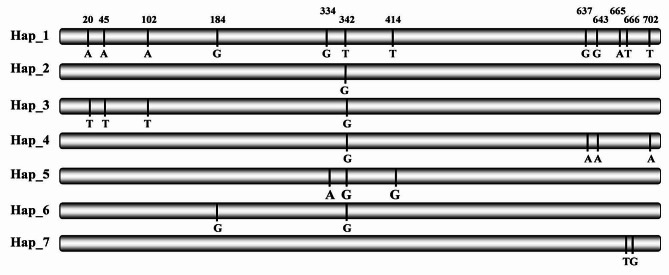
Sketch showing the *ATP6* mutation position in nucleotide haplotypes compared to nucleotide sequence in Hap_1

A total of 12 variable sites were detected in the 139 full nucleotide sequences, representing 1–9 sites from each of the seven populations (Table 1). These variable sites formed seven nucleotide haplotypes which were deduced into six isoforms (Fig. 1; Tables 2 and 3). All sequence variations were generated by point mutations. Nucleotide diversity in the seven populations ranged from 0.00019 to 0.00092, with a total diversity of 0.00092 in the pooled population (Table 1). Haplotype diversity in the seven field populations ranged from 0.133 to 0.525, with a total diversity of 0.539 in the combined population (Table 1). The highest nucleotide diversity and the richest segregating sites were found in the *P. infestans* population sampled from Xiapu while the highest haplotype diversity was found in the population sampled from Guizhou, respectively (Table 1). However, the pathogen population sampled from Yunnan and Ningxia, two sites at the highest altitude, possessed the lowest number of segregating sites, the least haplotype diversity, and the least nucleotide diversity.

**Table 2:** The nucleotide positions and mutation types of the 7 nucleotide haplotypes of *ATP6*

			1	1	3	3	4	6	6	6	6	7
	2	4	0	8	3	4	1	3	4	6	6	0
	0*	5	2	4	4	2	4	7	3	5	6	2
Hap_1	A	A	A	G	G	T	T	G	G	A	T	T
Hap_2	.	.	.	.	.	G	.	.	.	.	.	.
Hap_3	T	T	T	.	.	G	.	.	.	.	.	.
Hap_4	.	.	.	.	.	G	.	A	A	.	.	A
Hap_5	.	.	.	.	A	G	G	.	.	.	.	.
Hap_6	.	.	.	A	.	G	.	.	.	.	.	.
Hap_7	.	.	.	.	.	.	.	.	.	T	G	.

*Nucleotide position in the gene, Dot represents the same nucleotide as Hap_1

**Table 3:** The frequency distribution of *ATP6* nucleotide haplotypes in each of seven populations

Haplotypes	Fuzhou	Xiapu	Gansu	Guizhou	Ningxia	Guangxi	Yunnan	Pooled
Hap_1	0.238	0.067	0.750	0.563	0.933	0.455	0.933	0.496
Hap_2	0.762	0.833	0.150	0.438	0.067	0.545	0.067	0.468
Others*	0.000	0.100	0.100	0.000	0.000	0.000	0.000	0.036
Sum	1.000	1.000	1.000	1.000	1.000	1.000	1.000	1.000

* Hap_3, 4, 5, 6 and 7 were merged, Haplotypes detected only once are combined into others. The *P* value of chi-square test is 0.001

### Population genetic structure of *ATP6* gene

**Fig. 2:**
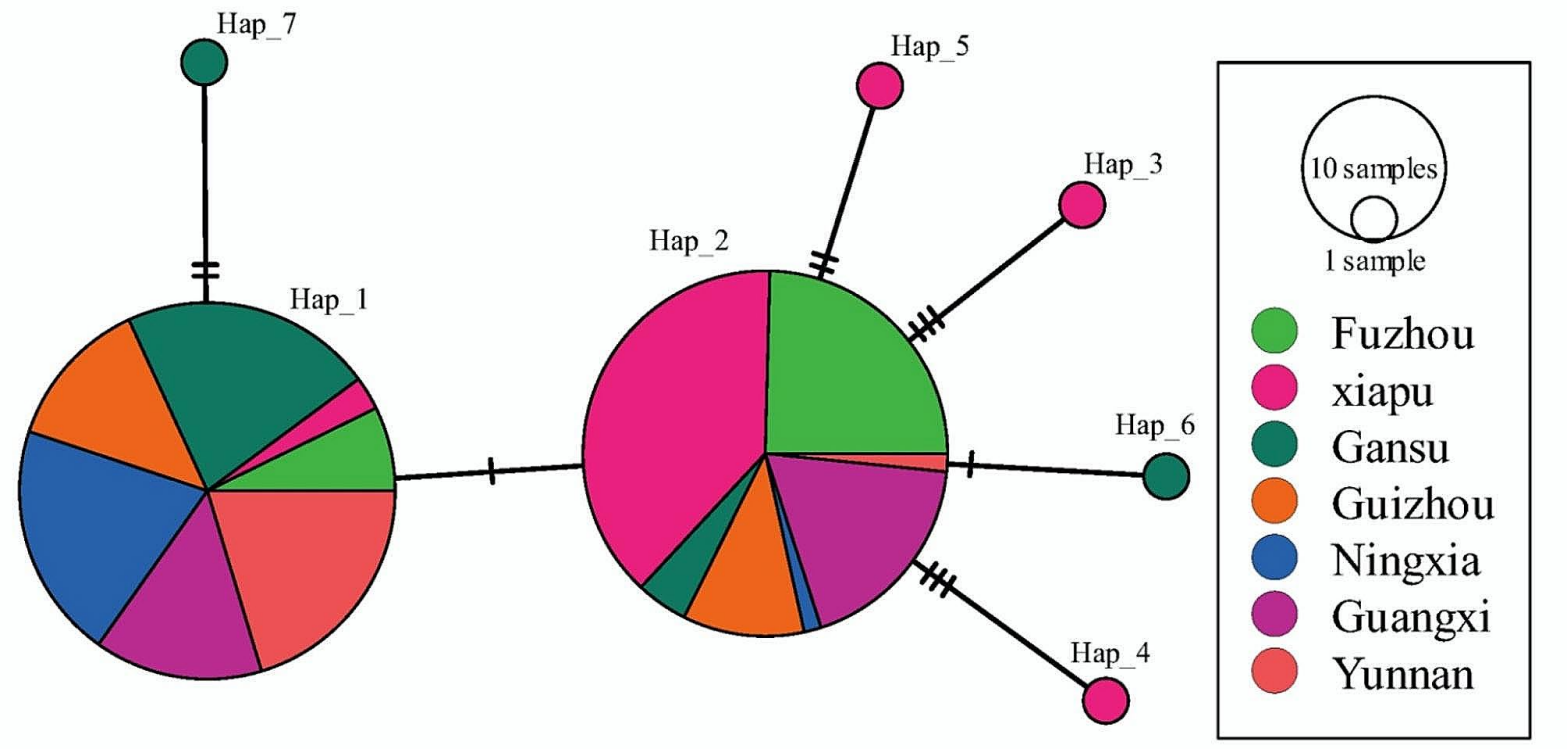
Haplotypes network of *ATP6* gene generated by PopArt 1.7. The network was constructed by a maximum parsimony approach. Colors represent geographic origins (populations) of the haplotypes and circle size represent the frequency of the haplotypes in each subpopulation

Among the seven nucleotide haplotypes, Hap_1 and Hap_2, which are translated into the same isoform, were predominant in all seven populations (Table 2), with a frequency ranging from 0.067 to 0.933 and 0.067–0.833 respectively in the individual populations (Table 3) and of 0.496 and 0.468 in the combined population, respectively. Other haplotypes, each deduced into different isoform, were detected only once and were private to each population (Fig. 2).

The pathogen populations from different locations varied significantly in the frequency of nucleotide haplotypes (Table 3) by the homogeneity test (*p* = 0.001). The hierarchical participation of genetic differentiation (F_ST_) in the SSR marker loci of the pathogen samples was 0.079 for among populations, 0.028 for among synonymous mutations and 0.031 for within the synonymous mutations with the total F_ST_ of 0.130. There was no difference in genetic differentiation among and within the synonymous mutations.

### Haplotype network and genetic distance

A network analysis revealed that most of the haplotypes were two mutation steps away from each other (Fig. 2). The majority of rare haplotypes were associated with one of the two major haplotypes Hap_1 and Hap_2. One minor haplotype (Hap_7) was more closely associated with Hap_1 than Hap_2, while four other haplotypes (Hap_3, Hap_4, Hap_5 and Hap_6) were more closely related to Hap_2 than Hap_1. Hap_7 was two mutation steps away from Hap_1. Hap_3 and Hap_4 were three mutation steps away from Hap_2, while Hap_5 and Hap_6 were two and one mutation steps away from Hap_2, respectively (Fig. 2).

### Comparison of metabolic rate, aggressiveness, temperature sensitivity and fungicide tolerance among the haplotypes

**Fig. 3:**
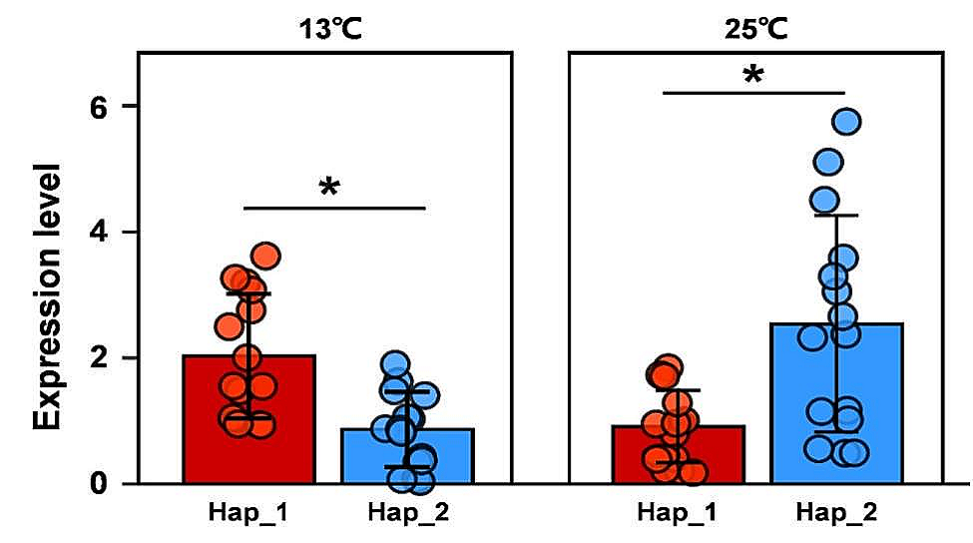
Expression level of *ATP6* gene in Hap_1 and Hap_2 under different temperatures. The experimental isolates were grown under 13 and 25 °C for eight days. Significant differences between Hap_1 and Hap_2 under each temperature are shown by * (*p* < 0.05)

Analysis of variance by general linear model (GLM) found “population”, “isolate” and “haplotype” contributed significantly (*p* < 0.05) to differences in metabolic rate, temperature sensitivity, fungicide tolerance and aggressiveness among the *P. infestans* isolates sampled from different locations. Least significant difference (LSD) analysis revealed that the same isoform deduced from different haplotypes, i.e., synonyms mutations, differed significant in all measurement of fitness components. Hap_1 and Hap_2 are translated into an identical amino acid isoform but the isoform deduced from Hap_1 haplotype showed a lower metabolic rate, tolerance to hot stress (25 °C), azoxystrobin sensitivity and aggressiveness but higher tolerance to cold stress (13 °C) than that deduced from Hap_2 (Table 4). The isoforms deduced from other haplotypes displayed lower measurements in all of the fitness components than the isoform deduced from Hap_2 (Table 4). The expression level of Hap_1 was significantly higher than that of Hap_2 at 13 °C but significantly lower than that of Hap_2 at 25 °C (Fig. 3).

**Table 4:** Least significant difference test for aggressiveness, metabolic rate, azoxystrobin tolerance and temperature sensitivity among isolates with Hap_1, Hap_2 and minor haplotypes in the 139 *P. infestans* isolates collected from potato in China

Haplotypes	Sample size	Metabolic rate (19 °C)	Temperature sensitivity		Fungicide (µg/mL)	Aggressiveness (cm^2^)
			13 °C	25 °C		
Hap_1	69	0.429^B^	0.389^A^	0.318^B^	0.606^B^	15.233^B^
Hap_2	65	0.480^A^	0.364^B^	0.372^A^	0.642^A^	19.038^A^
Others*	5	0.451^B^	0.357^B^	0.363^AB^	0.618^B^	16.971^B^

* Hap_3, 4, 5, 6 and 7 were merged, Haplotypes detected only once are combined into others. Fungicide susceptibility was calculated as the average growth rate at five concentrations of azoxystrobin (0.01, 0.05, 0.10, 0.13, and 0.15 µg/mL) relative to the that without fungicide supplementation

### Associations of *ATP6* haplotype frequencies in *P. infestans* populations with the ecological data of collection site

**Fig. 4:**
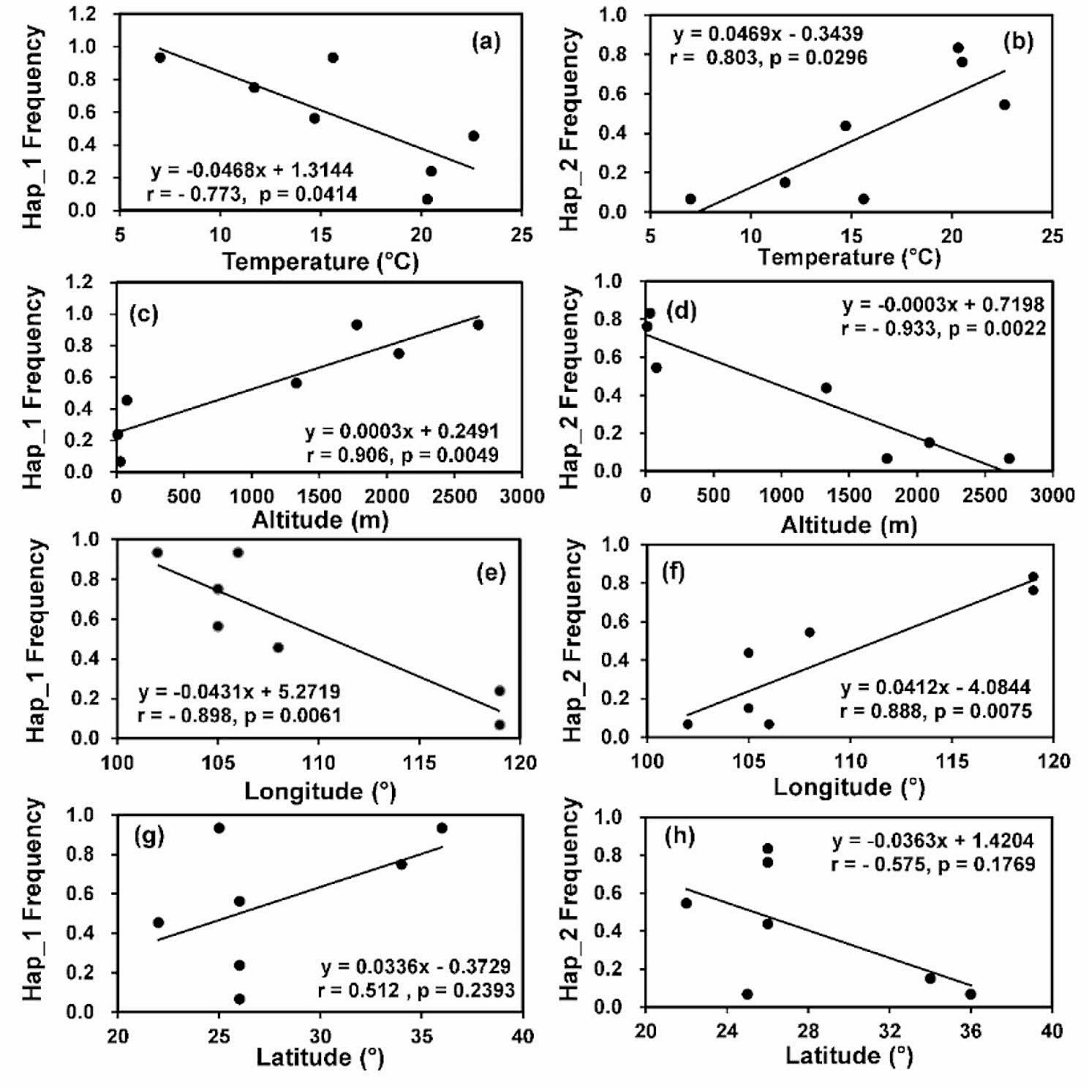
Correlation between the frequency of *ATP6* haplotypes in the seven *P. infestans* subpopulations and the annual mean temperature, altitude, longitude and latitude in the source population: (**a**) Hap_1 frequency and temperature; (**b**) Hap_2 frequency and temperature; (**c**) Hap_1frequency and altitude; (**d**) Hap_2 frequency and altitude; (**e**) Hap_1 frequency and longitude; (**f**) Hap_2 frequency and longitude; (**g**) Hap_1 frequency and latitude; (**h**) Hap_2 frequency and latitude

Spatial distribution of *ATP6* sequences was strongly affected by temperature and geographic landscape (Fig. 4). Hap_1 frequency was negatively associated with the annual mean temperature (*p* = 0.0414) and longitude (*p* = 0.0062) in the collection sites but positively associated with altitude (*p* = 0.0049). On the other hand, Hap_2 frequency was positively associated with the annual mean temperature in the collection sites (*p* = 0.0296) but negatively associated with altitude (*p* = 0.0022).

### UPGMA and PCA analyses

**Fig. 5:**
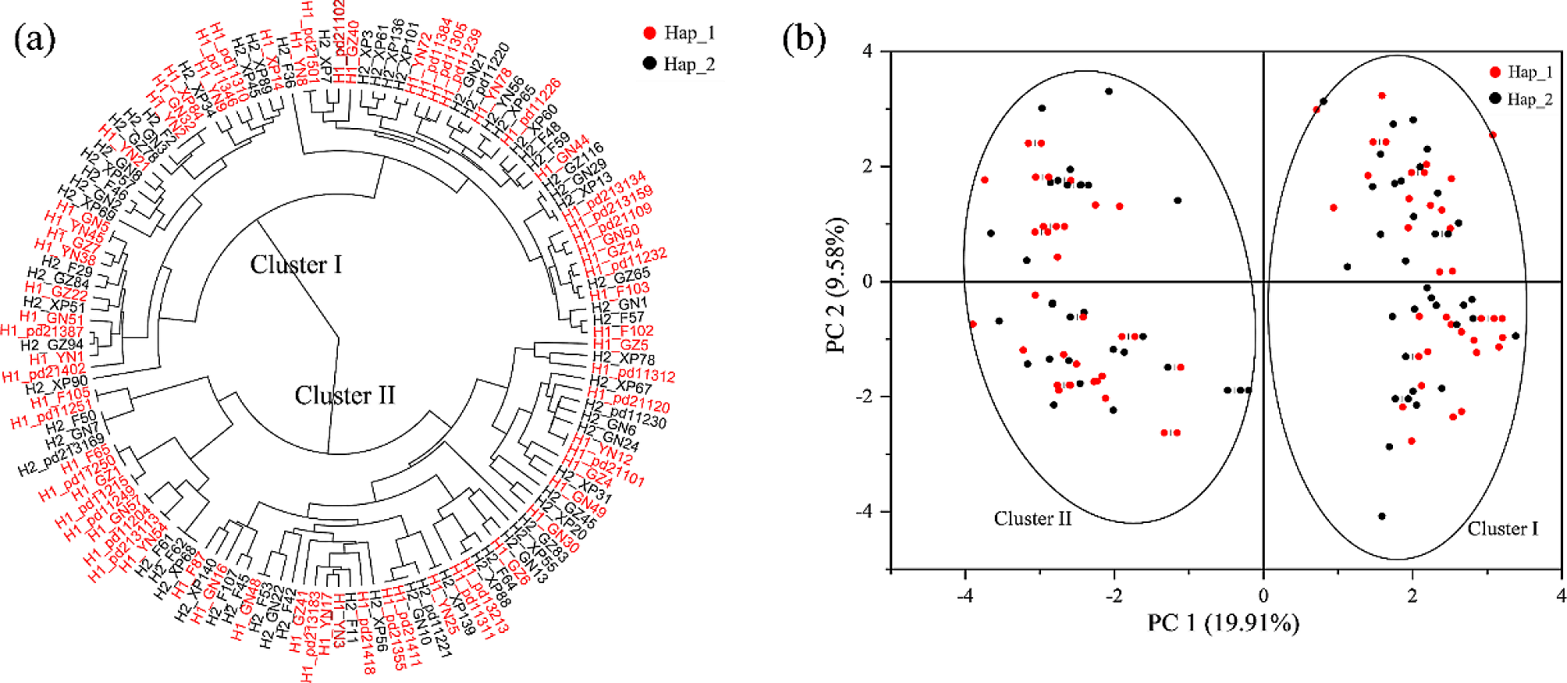
Dendrogram and Principal Component Analysis (PCA) of *Phytophthora infestans* isolates with Hap_1 and Hap_2_of *ATP6* sequences

Both the UPGMA and PCA analyses clustered into two groups (Fig. 5). Cluster I contained 36 isolates of Hap_1 and 34 isolates of Hap_2. On the other hand Cluster II contained 33 and 31isolates of Hap_1 and Hap_2, respectively. The Principal PCA analysis showed that component 1 and 2 explained 19.91% and 9.58% of genetic variation, respectively (Fig. 5b).

## Discussion

Low genetic variation and significant variation in haplotype-associated fitness was found in the *ATP6* gene of *P. infestans*. Only two main synonymous nucleotide haplotypes were found among the 139 genotypes. Sequence alignment and haplotype network analysis show that point mutations are the only mechanism generating sequence variation and most sequences only have 1–2 mutation steps away from each other. Furthermore, quantitative analysis of functional traits reveals significant variations in fitness among the haplotypes, consistent with a current report in yeast using ∼ 8,500 synonymous mutants generated from > 20 endogenous genes [69]. The conserved evolution, together with significant difference in the fitness components among the haplotypes suggests the *ATP6* is under strong natural selection to purge its genetic variation, consistent with the theoretical expectation for genome essential to maintain basic cellular functions [70]. The conserved evolution in housekeeping genes has been reported in the concerned pathogen [71] as well as other species across kingdoms [72–74].

In a spatial scale, conserved evolution associated with natural selection for similar mutants of housekeeping genes across geographic landscapes reduces their population differentiation relative to neutral markers [75]. Unexpectedly, we found non-random distribution of *ATP6* haplotypes across geographic gradients, suggesting that the *ATP6* gene may involve in local adaptation to ecological notches in addition to its primary role in cellular survival and proliferation. Considering the complexity of eco-composition and type of approaches we used in the study, we are unable to unequivocally identify the ecological factors determining the observed adaptation, but we believe that temperature should be one of the candidate drivers. This argument is aligned with documented results in other species [76, 77] and the observed associations of frequency in *ATP6* haplotypes with local air temperature in the current study. The mirrored associations of *ATP6* haplotypes with temperatures and altitudes of source populations may also reflect the importance of temperature in the adaptation of *ATP6* gene because air temperature always decreases as altitude increases [78]. This hypothesis is also aligned with expectations given the regulative effects of temperature on biological, genetic, and eco-evolutionary processes of species [79–81] and on *ATP6* activity to generate adequate bioenergy for cells. Like altitude, latitude is usually negatively associated with temperature [82]. Interestingly, we found the spatial distribution of *ATP6* haplotype was only marginally associated with latitudes but significantly associated with longitudes in the source population, further suggesting that multiple ecological factors may contribute to the local adaptation of the *ATP6* gene as proposed above. Understatedly, regression analysis usually cannot resolve the causal and resultant parameters. But in our case, the consideration of *ATP6* haplotypes as the causal parameter affecting air temperature in the source populations can be excluded without doubt.

Hap_1 and Hap_2 were the most common haplotypes and translated into the same isoform. However, the two haplotypes varied significantly in all four function traits (metabolic rate, temperature sensitivity, aggressiveness and fungicide resistance) evaluated. These four functional traits constitute the most important fitness components that can strongly influence pathogen growth (associated with metabolic rate), disease epidemics (associated with aggressiveness) and management efficacy (associated with fungicide resistance) in the future climatic conditions associated with global warming (associated with temperature sensitivity). Most *P. infestans* worldwide reproduce asexually, generating populations dominated by one or a few clonal lineages [48, 83]. Genotypes within clonal lineages often contain minor sequence differences caused by mutations, but substantial sequence differences may exist between clonal lineages, leading to higher population differentiation between clonal lineages than within clonal lineages. Hap_1 and Hap_2 may belong to different clonal lineages, and the difference in fitness between the two haplotypes may be associated with this clonal feature. However, we found no difference in population differentiation among and within the synonymous mutations (see result in population genetic structure) and similar members of the two haplotypes presenting in each cluster (Fig. 5), suggesting that isolates in the synonymous mutation groups do not associate with particular clonal lineages and our observations of phenotypic difference between the two synonymous mutations may not be caused by the clonal lineage. The fitness components were estimated by taking an average of 134 isolates with 69 for Hap_1 and 65 for Hap_2, respectively. The sample size included is larger than many similar studies to infer fitness polymorphisms of synonymous mutations [22, 84], which allows us to largely remove background effects. Furthermore, clonal structure can lead to higher genetic differentiation among than within clonal lineages. More importantly, we found significant differences in allelic composition and mating types among isolates within the same *ATP6* haplotype (Table S1). For example, 8 out of 16 alleles differ between isolates YN38 and pd11249 in Hap_1. Likewise, 6 of 16 alleles differ between isolates XP67 and F107 in Hap_2. On the other hand, only 3 out of 16 alleles differ between pd11249 in Hap_1 and XP67 in Hap_2. Although not completely ruled out, these results further suggest that isolates within the same *ATP6* haplotype may not originate from the same clone and that differences in aggressiveness, fungicide resistance and temperature sensitivity between Hap_1 and Hap_2 may not be due to fitness variation between clonal lineages. *Phytophthora infestans* can reproduce both sexually and asexually. Self-fertile isolates have emerged and become dominant in some of the regions [61] where isolates were included in the study, providing the opportunity to generate distinct genotypes in the pathogen populations. Therefore, we hypothesizes synonymous mutations may also contribute to the difference in fitness between Hap_1 and Hap_2, consistent with recent results which show that these mutations are not always selectively neutral [85]. In biology, we usually consider the genetics in tern of DNA sequences, but this is often not the case. RNA editing is ubiquitous in all organisms, resulting in discrepancies between the DNA sequence and the actual RNA sequence being translated into proteins, and may also be specific in genome context [86–89]. Codon usage bias can also regulate protein expression [17, 23] and affect the biological function of genes with synonymous mutations.

Quantifying the impacts of a synonymous mutation on the fitness of species is particularly rare. Empirical evaluation indicates most of synonymous mutations have, if any, only small impact on fitness [90], but substantial fitness benefit/penalty has also been documented in some empirical studies [21, 22, 91]. For example, 7–9% of fitness differences were found in the synonymous mutations of *Pseudomonas fluorescens gtsB* gene [92]. In our study, fitness difference between Hap_1 and Hap_2 ranged from a low of 5.6% in fungicide resistance to a high of 20.0% in aggressiveness with an average of 11.4%. Considering only one base difference between the two haplotypes, the current estimates of fitness benefit/penalty associated with the synonymous mutation are surprisedly large and should be interpreted cautiously.

It is well-known that synonymous codons are unevenly used [93]. GC-ending synonymous codons appear more frequently in genomes than the neutral expectation [94]. Several theories have been established to explain the preference for GC-ending codons. One theory assumes that GC-ending synonymous codons demonstrate a higher thermal stability compared with their counterparts ending with AT base pairs. This differentiation in thermal features arises from the stronger stacking interaction between GC bases due to triple hydrogen bond compared with a double hydrogen bond between the pair bases in AT ending codons [95] and can have critical influence on thermal preference of organisms [96, 97]. This theory may partially explain the contrary thermal adaptation between Hap_1 and Hap_2. Thermal stability in the GC-ending Hap_2 is expected to be higher, contributing to its better fitness and higher abundance in hot environment. On the other hand, replacing GC-ending by AT-ending synonymous codons in Hap_1 reduces its thermal stability confers the better cold tolerance of the haplotypes and its negative association with air temperature. Consistent with these theories and observations, we found that expression of the *ATP6* gene was significantly higher in Hap_2 isolates when grown at high temperature (25 °C) while Hap_1 isolates grown at low temperature (13 °C) showed significantly higher *ATP6* gene expression (Fig. 3).

However, the thermal stability theory alone may not explain the significant difference in aggressiveness, fungicide resistance and intrinsic metabolic rate between Hap_1 and Hap_2. It has been documented that GC-ending codons are decoded at higher rates during mRNA translation and protein elongation [98] due to higher availability of the transfer RNA (tRNA). Consequently, GC-ending synonymous codons have higher codon adaptation index [99] or tRNA adaptation index [100] than their AT-ending counterparts. This polymorphism in transcriptional efficiency between GC- and AT-ending codons may enhance the overall fitness of Hap_2, including its higher aggressiveness, fungicide resistance and metabolic rate than Hap_1.

## Conclusions

Our results reveal that synonymous mutations might influence the development of many functional traits and, therefore, play an important role in the adaptive evolution of the pathogen. GC-ending codons may perform better than AT-ending codons, particularly under hot conditions, possibly due to higher thermostatic and transcriptional efficiency in the former group. The finding provides important insight for understanding the evolutionary pattern and consequence of host-pathogen-environment interaction and worthy of attracting more attention in the field of plant pathology. However, our results were drawn from a comparative population analysis between a pair of synonymous mutations in DNA sequences. Although we currently lack RNA sequence data for the strains, we recognize the importance of future investigations to explore the connection between codon usage bias and translation rate based on RNA sequences, considering the prevalence of RNA editing reported in the literature [86–89]. Further studies with a series of synonymous mutations (preferably generated by gene-editing from the same ancestry genotypes, i.e., same genetic background) from various functional genes such as effectors, fungicide targeting sites etc. are also required to advance our understanding of this important biological and evolutionary question.

### Electronic supplementary material

Below is the link to the electronic supplementary material.

Supplementary Material 1

## Data Availability

The *ATP6* haplotype sequence data presented in the study were deposited in NCBI with accession numbers of KX999558– KX999649, KX999653– KX999699, FAFU science & technology innovation fund, KFB23031A.

## Abbreviations

RGR: Relative growth rate
SSR: Simple sequence repeats
AUDPC: Area under the disease progress curve
ANOVA: Analysis of variance
LSD: Least significant differences
GLM: General linear model
