# Supplementary material for: Fitness difference between two synonymous mutations of Phytophthora infestans ATP6 gene
Source: BMC Ecol Evol. 2024 Mar 18;24:36. doi: 10.1186/s12862-024-02223-4 (PMC10946160; doi:10.1186/s12862-024-02223-4)
Supplement: Supplementary file 1 — Supplementary Material 1 [file 12862_2024_2223_MOESM1_ESM.docx]

Table S1 List of ATP6 haplotypes and their SSR composition, virulence factor (race) and mating types

| **ATP6** | **Isolate** | **SSR marker** | | | | | | | | | | | | | | | | | **Virulence factor** | | | | | | | | | | | | | | | | | | | | | | | | **MT*** | |
| --- | --- | --- | --- | --- | --- | --- | --- | --- | --- | --- | --- | --- | --- | --- | --- | --- | --- | --- | --- | --- | --- | --- | --- | --- | --- | --- | --- | --- | --- | --- | --- | --- | --- | --- | --- | --- | --- | --- | --- | --- | --- | --- | --- | --- |
|  |  | **G11** | | **pi56** | | **pi33** | | **pi04** | | **pi89** | | **pi4B** | | **pi02** | | **pi16** | | | **R1** | | **R2** | | **R3** | | **R4** | | **R5** | | **R6** | | **R7** | | **R8** | | **R9** | | **R10** | | **R11** | | **r** | |  |  |
| Hap_1 | pd11249 | 0 | 0 | 0 | 1 | 0 | 0 | 0 | 3 | 0 | 1 | 0 | 3 | 0 | 3 | | 1 | 2 | | 1 | | 0 | | 1 | | 1 | | 0 | | 1 | | 1 | | 1 | | 0 | | 1 | | 0 | | 1 | | 1 |
| Hap_1 | XP14 | 3 | 5 | 0 | 1 | 0 | 0 | 0 | 3 | 0 | 0 | 1 | 3 | 0 | 3 | | 1 | 2 | | 1 | | 0 | | 1 | | 1 | | 1 | | 0 | | 1 | | 0 | | 0 | | 1 | | 1 | | 1 | | 3 |
| Hap_1 | XP84 | 3 | 5 | 0 | 1 | 0 | 0 | 0 | 3 | 0 | 0 | 1 | 3 | 0 | 3 | | 1 | 2 | | 1 | | 1 | | 0 | | 0 | | 1 | | 0 | | 0 | | 0 | | 0 | | 0 | | 1 | | 1 | | 3 |
| Hap_1 | GN5 | 5 | 5 | 1 | 1 | 0 | 0 | 0 | 3 | 0 | 0 | 1 | 3 | 0 | 3 | | 1 | 2 | | 1 | | 0 | | 1 | | 1 | | 1 | | 0 | | 1 | | 0 | | 1 | | 0 | | 1 | | 1 | | 3 |
| Hap_1 | F102 | 0 | 5 | 0 | 1 | 0 | 0 | 0 | 4 | 0 | 0 | 1 | 3 | 0 | 3 | | 1 | 2 | | 0 | | 0 | | 0 | | 0 | | 0 | | 0 | | 1 | | 0 | | 0 | | 0 | | 1 | | 1 | | 3 |
| Hap_1 | F103 | 0 | 4 | 0 | 1 | 0 | 0 | 0 | 3 | 0 | 0 | 1 | 3 | 0 | 3 | | 1 | 2 | | 1 | | 0 | | 0 | | 0 | | 0 | | 0 | | 0 | | 0 | | 0 | | 0 | | 1 | | 1 | | 3 |
| Hap_1 | F105 | 0 | 0 | 0 | 1 | 0 | 0 | 0 | 3 | 0 | 1 | 1 | 3 | 0 | 3 | | 1 | 2 | | 0 | | 0 | | 1 | | 0 | | 0 | | 0 | | 0 | | 0 | | 0 | | 0 | | 0 | | 1 | | 1 |
| Hap_1 | F87 | 1 | 0 | 0 | 1 | 0 | 0 | 0 | 3 | 0 | 1 | 0 | 3 | 0 | 3 | | 1 | 2 | | 0 | | 0 | | 1 | | 0 | | 0 | | 1 | | 1 | | 0 | | 0 | | 0 | | 0 | | 1 | | 1 |
| Hap_1 | F65 | 1 | 0 | 0 | 1 | 0 | 0 | 0 | 3 | 0 | 1 | 0 | 3 | 0 | 3 | | 1 | 2 | | 1 | | 0 | | 1 | | 1 | | 0 | | 1 | | 1 | | 1 | | 0 | | 1 | | 0 | | 1 | | 1 |
| Hap_1 | GN32 | 3 | 5 | 0 | 1 | 0 | 0 | 0 | 3 | 0 | 0 | 1 | 3 | 0 | 3 | | 1 | 2 | | 1 | | 1 | | 0 | | 0 | | 1 | | 0 | | 0 | | 0 | | 0 | | 0 | | 1 | | 1 | | 3 |
| Hap_1 | YN12 | 0 | 2 | 0 | 1 | 0 | 0 | 0 | 3 | 0 | 3 | 0 | 3 | 0 | 3 | | 1 | 2 | | 0 | | 0 | | 0 | | 1 | | 0 | | 0 | | 1 | | 1 | | 0 | | 0 | | 0 | | 1 | | 1 |
| Hap_1 | YN21 | 2 | 5 | 1 | 1 | 0 | 0 | 0 | 3 | 0 | 0 | 1 | 3 | 0 | 3 | | 1 | 2 | | 0 | | 0 | | 0 | | 1 | | 1 | | 0 | | 0 | | 1 | | 0 | | 1 | | 1 | | 1 | | 3 |
| Hap_1 | GZ5 | 1 | 0 | 0 | 1 | 0 | 0 | 0 | 3 | 0 | 3 | 0 | 3 | 0 | 3 | | 1 | 2 | | 0 | | 0 | | 1 | | 0 | | 0 | | 1 | | 1 | | 1 | | 1 | | 0 | | 0 | | 1 | | 1 |
| Hap_1 | YN1 | 4 | 5 | 1 | 1 | 0 | 0 | 0 | 3 | 0 | 0 | 1 | 3 | 0 | 3 | | 1 | 2 | | 0 | | 0 | | 0 | | 0 | | 0 | | 0 | | 1 | | 0 | | 0 | | 1 | | 1 | | 1 | | 3 |
| Hap_1 | pd21402 | 5 | 5 | 1 | 1 | 0 | 0 | 0 | 3 | 0 | 0 | 2 | 3 | 0 | 3 | | 1 | 2 | | 1 | | 0 | | 1 | | 1 | | 0 | | 1 | | 0 | | 0 | | 0 | | 1 | | 1 | | 1 | | 3 |
| Hap_1 | GZ14 | 0 | 5 | 0 | 1 | 0 | 0 | 0 | 4 | 0 | 0 | 1 | 3 | 0 | 3 | | 1 | 2 | | 1 | | 0 | | 0 | | 0 | | 0 | | 0 | | 0 | | 0 | | 0 | | 0 | | 1 | | 1 | | 3 |
| Hap_1 | pd13213 | 0 | 3 | 0 | 1 | 0 | 0 | 0 | 3 | 0 | 1 | 0 | 3 | 0 | 3 | | 1 | 2 | | 1 | | 0 | | 1 | | 1 | | 0 | | 1 | | 1 | | 1 | | 1 | | 1 | | 0 | | 1 | | 1 |
| Hap_1 | pd21120 | 0 | 2 | 0 | 1 | 0 | 0 | 0 | 3 | 0 | 3 | 0 | 3 | 0 | 3 | | 1 | 2 | | 0 | | 0 | | 0 | | 1 | | 0 | | 1 | | 1 | | 0 | | 0 | | 0 | | 0 | | 1 | | 1 |
| Hap_1 | pd11215 | 0 | 0 | 0 | 1 | 0 | 0 | 0 | 3 | 0 | 1 | 0 | 3 | 0 | 3 | | 1 | 2 | | 1 | | 0 | | 1 | | 1 | | 0 | | 1 | | 1 | | 1 | | 0 | | 1 | | 0 | | 1 | | 1 |
| Hap_1 | pd11239 | 0 | 6 | 0 | 1 | 0 | 0 | 0 | 4 | 0 | 0 | 1 | 3 | 0 | 3 | | 1 | 2 | | 0 | | 0 | | 1 | | 1 | | 1 | | 0 | | 0 | | 0 | | 0 | | 0 | | 1 | | 1 | | 3 |
| Hap_1 | pd11312 | 1 | 1 | 0 | 1 | 0 | 0 | 0 | 3 | 0 | 3 | 0 | 3 | 0 | 3 | | 1 | 2 | | 1 | | 0 | | 1 | | 1 | | 0 | | 0 | | 1 | | 1 | | 0 | | 1 | | 0 | | 1 | | 1 |
| Hap_1 | pd11226 | 0 | 5 | 0 | 1 | 0 | 0 | 0 | 4 | 0 | 0 | 1 | 3 | 0 | 3 | | 1 | 2 | | 1 | | 0 | | 1 | | 1 | | 1 | | 0 | | 1 | | 0 | | 1 | | 0 | | 1 | | 1 | | 3 |
| Hap_1 | pd11384 | 0 | 5 | 0 | 1 | 0 | 0 | 0 | 3 | 0 | 0 | 2 | 3 | 0 | 3 | | 1 | 2 | | 0 | | 0 | | 1 | | 1 | | 1 | | 0 | | 0 | | 0 | | 0 | | 0 | | 1 | | 1 | | 3 |
| Hap_1 | pd11204 | 1 | 0 | 0 | 1 | 0 | 0 | 0 | 3 | 0 | 0 | 0 | 3 | 0 | 3 | | 1 | 2 | | 1 | | 1 | | 1 | | 1 | | 0 | | 1 | | 1 | | 1 | | 1 | | 0 | | 0 | | 1 | | 1 |
| Hap_1 | pd213159 | 0 | 5 | 0 | 1 | 0 | 0 | 0 | 3 | 0 | 0 | 2 | 3 | 0 | 3 | | 1 | 2 | | 1 | | 1 | | 0 | | 0 | | 1 | | 0 | | 0 | | 0 | | 0 | | 0 | | 1 | | 1 | | 3 |
| Hap_1 | pd21418 | 1 | 2 | 1 | 1 | 0 | 0 | 0 | 3 | 0 | 1 | 0 | 3 | 0 | 3 | | 1 | 2 | | 0 | | 0 | | 0 | | 0 | | 0 | | 1 | | 1 | | 1 | | 0 | | 0 | | 0 | | 1 | | 1 |
| Hap_1 | GN44 | 0 | 5 | 0 | 1 | 0 | 0 | 0 | 3 | 0 | 0 | 2 | 3 | 0 | 3 | | 1 | 2 | | 0 | | 0 | | 1 | | 0 | | 1 | | 0 | | 1 | | 0 | | 0 | | 1 | | 1 | | 1 | | 3 |
| Hap_1 | pd21501 | 0 | 5 | 1 | 1 | 0 | 0 | 0 | 3 | 0 | 0 | 1 | 3 | 0 | 3 | | 1 | 2 | | 1 | | 1 | | 1 | | 1 | | 1 | | 0 | | 1 | | 1 | | 0 | | 1 | | 1 | | 1 | | 3 |
| Hap_1 | GN49 | 1 | 2 | 1 | 1 | 0 | 0 | 0 | 3 | 0 | 3 | 0 | 3 | 0 | 3 | | 1 | 2 | | 0 | | 0 | | 1 | | 0 | | 0 | | 1 | | 1 | | 1 | | 0 | | 0 | | 0 | | 1 | | 1 |
| Hap_1 | pd11251 | 0 | 0 | 0 | 1 | 0 | 0 | 0 | 3 | 0 | 1 | 1 | 3 | 0 | 3 | | 1 | 2 | | 0 | | 0 | | 1 | | 0 | | 0 | | 0 | | 0 | | 0 | | 0 | | 0 | | 0 | | 1 | | 1 |
| Hap_1 | pd11311 | 1 | 3 | 1 | 1 | 0 | 0 | 0 | 3 | 0 | 1 | 0 | 3 | 0 | 3 | | 1 | 2 | | 1 | | 0 | | 1 | | 1 | | 0 | | 1 | | 1 | | 1 | | 0 | | 1 | | 0 | | 1 | | 1 |
| Hap_1 | pd11346 | 3 | 5 | 0 | 1 | 0 | 0 | 0 | 3 | 0 | 0 | 1 | 3 | 0 | 3 | | 1 | 2 | | 0 | | 0 | | 1 | | 1 | | 1 | | 0 | | 1 | | 1 | | 1 | | 1 | | 1 | | 1 | | 3 |
| Hap_1 | GZ1 | 1 | 0 | 0 | 1 | 0 | 0 | 0 | 3 | 0 | 1 | 0 | 3 | 0 | 3 | | 1 | 2 | | 1 | | 0 | | 1 | | 1 | | 0 | | 1 | | 1 | | 1 | | 0 | | 1 | | 0 | | 1 | | 1 |
| Hap_1 | GZ40 | 0 | 5 | 0 | 1 | 0 | 0 | 0 | 3 | 0 | 0 | 0 | 3 | 0 | 3 | | 1 | 2 | | 0 | | 0 | | 1 | | 1 | | 1 | | 0 | | 0 | | 1 | | 1 | | 1 | | 1 | | 1 | | 3 |
| Hap_1 | GZ41 | 1 | 1 | 1 | 1 | 0 | 0 | 0 | 3 | 0 | 1 | 0 | 3 | 0 | 3 | | 1 | 2 | | 0 | | 0 | | 1 | | 0 | | 0 | | 1 | | 1 | | 1 | | 1 | | 0 | | 0 | | 1 | | 1 |
| Hap_1 | pd21101 | 0 | 2 | 0 | 1 | 0 | 0 | 0 | 3 | 0 | 3 | 0 | 3 | 0 | 3 | | 1 | 2 | | 0 | | 0 | | 0 | | 1 | | 0 | | 0 | | 1 | | 1 | | 0 | | 0 | | 0 | | 1 | | 1 |
| Hap_1 | pd21102 | 0 | 5 | 0 | 1 | 0 | 0 | 0 | 3 | 0 | 0 | 0 | 3 | 0 | 3 | | 1 | 2 | | 1 | | 1 | | 1 | | 1 | | 1 | | 0 | | 1 | | 1 | | 1 | | 1 | | 1 | | 1 | | 3 |
| Hap_1 | pd21109 | 0 | 5 | 0 | 1 | 0 | 0 | 0 | 4 | 0 | 1 | 1 | 3 | 0 | 3 | | 1 | 2 | | 1 | | 1 | | 0 | | 0 | | 1 | | 0 | | 0 | | 0 | | 0 | | 0 | | 1 | | 1 | | 3 |
| Hap_1 | pd213113 | 1 | 0 | 0 | 1 | 0 | 0 | 0 | 3 | 0 | 1 | 0 | 3 | 0 | 3 | | 1 | 2 | | 1 | | 1 | | 1 | | 1 | | 0 | | 1 | | 1 | | 1 | | 1 | | 1 | | 0 | | 1 | | 1 |
| Hap_1 | pd213134 | 0 | 5 | 0 | 1 | 0 | 0 | 0 | 4 | 0 | 1 | 1 | 3 | 0 | 3 | | 1 | 2 | | 1 | | 0 | | 0 | | 0 | | 0 | | 0 | | 0 | | 1 | | 0 | | 1 | | 1 | | 1 | | 3 |
| Hap_1 | pd213183 | 1 | 2 | 0 | 1 | 0 | 0 | 0 | 3 | 0 | 1 | 0 | 3 | 0 | 3 | | 1 | 2 | | 0 | | 0 | | 1 | | 0 | | 0 | | 1 | | 1 | | 0 | | 0 | | 0 | | 0 | | 1 | | 1 |
| Hap_1 | pd21355 | 1 | 2 | 1 | 1 | 0 | 0 | 0 | 3 | 0 | 1 | 0 | 3 | 0 | 3 | | 1 | 2 | | 0 | | 0 | | 1 | | 1 | | 0 | | 0 | | 1 | | 0 | | 0 | | 0 | | 0 | | 1 | | 1 |
| Hap_1 | pd21387 | 5 | 5 | 1 | 1 | 0 | 0 | 0 | 3 | 0 | 0 | 1 | 3 | 0 | 3 | | 1 | 2 | | 0 | | 0 | | 0 | | 0 | | 0 | | 0 | | 1 | | 0 | | 0 | | 0 | | 1 | | 1 | | 3 |
| Hap_1 | pd21411 | 1 | 2 | 1 | 1 | 0 | 0 | 0 | 3 | 0 | 1 | 0 | 3 | 0 | 3 | | 1 | 2 | | 0 | | 0 | | 1 | | 1 | | 0 | | 0 | | 1 | | 0 | | 0 | | 0 | | 0 | | 1 | | 1 |
| Hap_1 | pd11310 | 3 | 5 | 0 | 1 | 0 | 0 | 0 | 3 | 0 | 0 | 1 | 3 | 0 | 3 | | 1 | 2 | | 1 | | 1 | | 1 | | 1 | | 1 | | 0 | | 1 | | 1 | | 1 | | 1 | | 1 | | 1 | | 3 |
| Hap_1 | YN17 | 1 | 2 | 0 | 1 | 0 | 0 | 0 | 3 | 0 | 1 | 0 | 3 | 0 | 3 | | 1 | 2 | | 0 | | 0 | | 1 | | 0 | | 0 | | 1 | | 1 | | 1 | | 0 | | 0 | | 0 | | 1 | | 1 |
| Hap_1 | YN25 | 1 | 2 | 1 | 1 | 0 | 0 | 0 | 3 | 0 | 1 | 0 | 3 | 0 | 3 | | 1 | 2 | | 0 | | 1 | | 1 | | 0 | | 0 | | 0 | | 0 | | 0 | | 0 | | 0 | | 0 | | 1 | | 1 |
| Hap_1 | YN45 | 5 | 5 | 1 | 1 | 0 | 0 | 0 | 3 | 0 | 0 | 1 | 3 | 0 | 3 | | 1 | 2 | | 1 | | 0 | | 1 | | 1 | | 1 | | 0 | | 1 | | 0 | | 1 | | 0 | | 1 | | 1 | | 3 |
| Hap_1 | YN54 | 1 | 0 | 0 | 1 | 0 | 0 | 0 | 3 | 0 | 1 | 0 | 3 | 0 | 3 | | 1 | 2 | | 1 | | 1 | | 1 | | 1 | | 0 | | 1 | | 1 | | 1 | | 1 | | 1 | | 0 | | 1 | | 1 |
| Hap_1 | YN72 | 1 | 5 | 0 | 1 | 0 | 0 | 0 | 4 | 0 | 0 | 1 | 3 | 0 | 3 | | 1 | 2 | | 1 | | 0 | | 1 | | 1 | | 1 | | 1 | | 0 | | 1 | | 0 | | 1 | | 1 | | 1 | | 3 |
| Hap_1 | YN78 | 1 | 5 | 0 | 1 | 0 | 0 | 0 | 4 | 0 | 0 | 1 | 3 | 0 | 3 | | 1 | 2 | | 1 | | 0 | | 1 | | 1 | | 1 | | 0 | | 1 | | 0 | | 0 | | 1 | | 1 | | 1 | | 3 |
| Hap_1 | GN30 | 1 | 2 | 1 | 1 | 0 | 0 | 0 | 3 | 0 | 4 | 0 | 3 | 0 | 3 | | 1 | 2 | | 1 | | 1 | | 1 | | 1 | | 0 | | 1 | | 1 | | 1 | | 1 | | 0 | | 0 | | 1 | | 1 |
| Hap_1 | GN48 | 0 | 2 | 0 | 1 | 0 | 0 | 0 | 3 | 0 | 1 | 1 | 3 | 0 | 3 | | 1 | 2 | | 0 | | 0 | | 1 | | 0 | | 0 | | 1 | | 1 | | 1 | | 1 | | 0 | | 0 | | 1 | | 1 |
| Hap_1 | GN16 | 1 | 0 | 0 | 1 | 0 | 0 | 0 | 3 | 0 | 1 | 0 | 3 | 0 | 3 | | 1 | 2 | | 0 | | 0 | | 1 | | 0 | | 0 | | 0 | | 0 | | 1 | | 0 | | 0 | | 0 | | 1 | | 1 |
| Hap_1 | GN57 | 1 | 0 | 0 | 1 | 0 | 0 | 0 | 3 | 0 | 0 | 0 | 3 | 0 | 3 | | 1 | 2 | | 1 | | 1 | | 1 | | 1 | | 0 | | 1 | | 1 | | 1 | | 1 | | 0 | | 0 | | 1 | | 1 |
| Hap_1 | GZ22 | 5 | 5 | 1 | 1 | 0 | 0 | 0 | 3 | 0 | 0 | 2 | 3 | 0 | 3 | | 1 | 2 | | 1 | | 1 | | 1 | | 1 | | 1 | | 0 | | 0 | | 1 | | 0 | | 1 | | 1 | | 1 | | 3 |
| Hap_1 | GZ4 | 1 | 2 | 1 | 1 | 0 | 0 | 0 | 3 | 0 | 3 | 0 | 3 | 0 | 3 | | 1 | 2 | | 0 | | 0 | | 0 | | 1 | | 0 | | 0 | | 1 | | 1 | | 0 | | 0 | | 0 | | 1 | | 1 |
| Hap_1 | GZ6 | 1 | 2 | 0 | 1 | 0 | 0 | 0 | 3 | 0 | 1 | 0 | 3 | 0 | 3 | | 1 | 2 | | 1 | | 1 | | 1 | | 1 | | 0 | | 0 | | 1 | | 1 | | 0 | | 1 | | 0 | | 1 | | 1 |
| Hap_1 | YN2 | 3 | 5 | 0 | 1 | 0 | 0 | 0 | 3 | 0 | 0 | 1 | 3 | 0 | 3 | | 1 | 2 | | 1 | | 1 | | 0 | | 0 | | 1 | | 0 | | 0 | | 0 | | 0 | | 0 | | 1 | | 1 | | 3 |
| Hap_1 | GN51 | 5 | 5 | 1 | 1 | 0 | 0 | 0 | 3 | 0 | 0 | 2 | 3 | 0 | 3 | | 1 | 2 | | 0 | | 0 | | 0 | | 0 | | 0 | | 0 | | 0 | | 1 | | 0 | | 1 | | 1 | | 1 | | 3 |
| Hap_1 | YN8 | 0 | 5 | 1 | 1 | 0 | 0 | 0 | 3 | 0 | 0 | 1 | 3 | 0 | 3 | | 1 | 2 | | 1 | | 1 | | 1 | | 1 | | 1 | | 0 | | 1 | | 1 | | 0 | | 1 | | 1 | | 1 | | 3 |
| Hap_1 | GN50 | 0 | 5 | 0 | 1 | 0 | 0 | 0 | 4 | 0 | 0 | 1 | 3 | 0 | 3 | | 1 | 2 | | 1 | | 1 | | 0 | | 0 | | 1 | | 0 | | 0 | | 0 | | 0 | | 0 | | 1 | | 1 | | 3 |
| Hap_1 | GZ7 | 5 | 5 | 1 | 1 | 0 | 0 | 0 | 3 | 0 | 0 | 1 | 3 | 0 | 3 | | 1 | 2 | | 0 | | 0 | | 1 | | 0 | | 1 | | 0 | | 1 | | 0 | | 0 | | 1 | | 1 | | 1 | | 3 |
| Hap_1 | YN3 | 1 | 2 | 0 | 1 | 0 | 0 | 0 | 3 | 0 | 1 | 0 | 3 | 0 | 3 | | 1 | 2 | | 0 | | 0 | | 1 | | 0 | | 0 | | 0 | | 1 | | 1 | | 0 | | 0 | | 0 | | 1 | | 1 |
| Hap_1 | pd11232 | 1 | 5 | 0 | 1 | 0 | 0 | 0 | 4 | 0 | 0 | 1 | 3 | 0 | 3 | | 1 | 2 | | 1 | | 0 | | 0 | | 0 | | 0 | | 0 | | 0 | | 0 | | 0 | | 0 | | 1 | | 1 | | 3 |
| Hap_1 | pd11250 | 1 | 0 | 0 | 1 | 0 | 0 | 0 | 3 | 0 | 1 | 0 | 3 | 0 | 3 | | 1 | 2 | | 1 | | 0 | | 1 | | 1 | | 0 | | 1 | | 1 | | 1 | | 0 | | 1 | | 0 | | 1 | | 1 |
| Hap_1 | pd11305 | 1 | 5 | 0 | 1 | 0 | 0 | 0 | 3 | 0 | 0 | 1 | 3 | 0 | 3 | | 1 | 2 | | 0 | | 0 | | 1 | | 1 | | 1 | | 0 | | 0 | | 0 | | 0 | | 0 | | 1 | | 1 | | 3 |
| Hap_1 | YN9 | 3 | 5 | 0 | 1 | 0 | 0 | 0 | 3 | 0 | 0 | 1 | 3 | 0 | 3 | | 1 | 2 | | 1 | | 1 | | 1 | | 1 | | 1 | | 1 | | 0 | | 0 | | 0 | | 1 | | 1 | | 1 | | 3 |
| Hap_1 | YN38 | 5 | 5 | 1 | 1 | 0 | 0 | 0 | 3 | 0 | 0 | 1 | 3 | 0 | 3 | | 1 | 2 | | 1 | | 0 | | 1 | | 1 | | 1 | | 0 | | 1 | | 0 | | 0 | | 1 | | 1 | | 1 | | 3 |
| Hap_2 | XP67 | 0 | 2 | 0 | 1 | 0 | 0 | 0 | 3 | 0 | 3 | 0 | 3 | 0 | 3 | | 1 | 2 | | 1 | | 0 | | 1 | | 1 | | 0 | | 1 | | 1 | | 1 | | 1 | | 0 | | 0 | | 1 | | 1 |
| Hap_2 | F57 | 0 | 5 | 0 | 1 | 0 | 0 | 0 | 4 | 0 | 1 | 1 | 3 | 0 | 3 | | 1 | 2 | | 0 | | 0 | | 0 | | 0 | | 0 | | 0 | | 1 | | 0 | | 0 | | 0 | | 1 | | 1 | | 3 |
| Hap_2 | XP57 | 2 | 5 | 1 | 1 | 0 | 0 | 0 | 3 | 0 | 0 | 1 | 3 | 0 | 3 | | 1 | 2 | | 1 | | 1 | | 1 | | 1 | | 1 | | 0 | | 0 | | 1 | | 0 | | 1 | | 1 | | 1 | | 3 |
| Hap_2 | XP89 | 3 | 5 | 0 | 1 | 0 | 0 | 0 | 3 | 0 | 0 | 1 | 3 | 0 | 3 | | 1 | 2 | | 0 | | 0 | | 1 | | 0 | | 1 | | 0 | | 1 | | 0 | | 0 | | 1 | | 1 | | 1 | | 3 |
| Hap_2 | F2 | 3 | 5 | 0 | 1 | 0 | 0 | 0 | 3 | 0 | 0 | 1 | 3 | 0 | 3 | | 1 | 2 | | 1 | | 1 | | 0 | | 0 | | 1 | | 0 | | 0 | | 0 | | 0 | | 0 | | 1 | | 1 | | 3 |
| Hap_2 | XP88 | 0 | 3 | 0 | 1 | 0 | 0 | 0 | 3 | 0 | 1 | 0 | 3 | 0 | 3 | | 1 | 2 | | 1 | | 0 | | 1 | | 1 | | 0 | | 1 | | 1 | | 1 | | 1 | | 1 | | 0 | | 1 | | 1 |
| Hap_2 | XP139 | 1 | 2 | 1 | 1 | 0 | 0 | 0 | 3 | 0 | 1 | 0 | 3 | 0 | 3 | | 1 | 2 | | 0 | | 1 | | 1 | | 0 | | 0 | | 0 | | 0 | | 0 | | 0 | | 0 | | 0 | | 1 | | 1 |
| Hap_2 | XP3 | 0 | 6 | 0 | 1 | 0 | 0 | 0 | 4 | 0 | 0 | 1 | 3 | 0 | 3 | | 1 | 2 | | 1 | | 0 | | 1 | | 1 | | 0 | | 1 | | 0 | | 0 | | 0 | | 1 | | 1 | | 1 | | 3 |
| Hap_2 | XP45 | 3 | 5 | 0 | 1 | 0 | 0 | 0 | 3 | 0 | 0 | 1 | 3 | 0 | 3 | | 1 | 2 | | 0 | | 0 | | 1 | | 1 | | 1 | | 0 | | 0 | | 0 | | 0 | | 0 | | 1 | | 1 | | 3 |
| Hap_2 | XP55 | 1 | 2 | 1 | 1 | 0 | 0 | 0 | 3 | 0 | 1 | 0 | 3 | 0 | 3 | | 1 | 2 | | 1 | | 1 | | 1 | | 1 | | 1 | | 1 | | 1 | | 1 | | 1 | | 1 | | 0 | | 1 | | 1 |
| Hap_2 | XP69 | 2 | 5 | 1 | 1 | 0 | 0 | 0 | 3 | 0 | 0 | 1 | 3 | 0 | 3 | | 1 | 2 | | 0 | | 0 | | 1 | | 0 | | 1 | | 0 | | 1 | | 0 | | 0 | | 1 | | 1 | | 1 | | 3 |
| Hap_2 | F11 | 1 | 2 | 0 | 1 | 0 | 0 | 0 | 3 | 0 | 1 | 0 | 3 | 0 | 3 | | 1 | 2 | | 0 | | 0 | | 1 | | 1 | | 0 | | 1 | | 1 | | 1 | | 0 | | 0 | | 0 | | 1 | | 1 |
| Hap_2 | F48 | 1 | 5 | 0 | 1 | 0 | 0 | 0 | 3 | 0 | 0 | 1 | 3 | 0 | 3 | | 1 | 2 | | 0 | | 0 | | 1 | | 0 | | 1 | | 0 | | 1 | | 0 | | 0 | | 1 | | 1 | | 1 | | 3 |
| Hap_2 | F29 | 5 | 5 | 1 | 1 | 0 | 0 | 0 | 3 | 0 | 0 | 1 | 3 | 0 | 3 | | 1 | 2 | | 0 | | 0 | | 1 | | 1 | | 1 | | 0 | | 0 | | 1 | | 1 | | 1 | | 1 | | 1 | | 3 |
| Hap_2 | F36 | 0 | 6 | 0 | 1 | 0 | 0 | 0 | 4 | 0 | 0 | 1 | 3 | 0 | 3 | | 1 | 2 | | 1 | | 0 | | 1 | | 1 | | 0 | | 1 | | 0 | | 0 | | 0 | | 1 | | 1 | | 1 | | 1 |
| Hap_2 | F53 | 0 | 2 | 0 | 1 | 0 | 0 | 0 | 3 | 0 | 1 | 1 | 3 | 0 | 3 | | 1 | 2 | | 0 | | 0 | | 1 | | 0 | | 0 | | 1 | | 1 | | 1 | | 1 | | 0 | | 0 | | 1 | | 1 |
| Hap_2 | XP101 | 1 | 5 | 0 | 1 | 0 | 0 | 0 | 3 | 0 | 0 | 1 | 3 | 0 | 3 | | 1 | 2 | | 1 | | 0 | | 1 | | 1 | | 1 | | 1 | | 0 | | 1 | | 0 | | 1 | | 1 | | 1 | | 3 |
| Hap_2 | XP13 | 0 | 5 | 0 | 1 | 0 | 0 | 0 | 4 | 0 | 1 | 1 | 3 | 0 | 3 | | 1 | 2 | | 1 | | 0 | | 0 | | 0 | | 0 | | 0 | | 0 | | 1 | | 0 | | 1 | | 1 | | 1 | | 3 |
| Hap_2 | GN1 | 0 | 4 | 0 | 1 | 0 | 0 | 0 | 3 | 0 | 0 | 1 | 3 | 0 | 3 | | 1 | 2 | | 0 | | 0 | | 0 | | 0 | | 0 | | 0 | | 1 | | 0 | | 0 | | 0 | | 1 | | 0 | | 3 |
| Hap_2 | GN22 | 0 | 2 | 0 | 1 | 0 | 0 | 0 | 3 | 0 | 1 | 1 | 3 | 0 | 3 | | 1 | 2 | | 0 | | 0 | | 1 | | 1 | | 0 | | 1 | | 1 | | 1 | | 0 | | 0 | | 0 | | 1 | | 1 |
| Hap_2 | GN2 | 2 | 5 | 0 | 1 | 0 | 0 | 0 | 4 | 0 | 0 | 1 | 3 | 0 | 3 | | 1 | 2 | | 0 | | 0 | | 0 | | 1 | | 1 | | 0 | | 0 | | 1 | | 0 | | 1 | | 1 | | 1 | | 3 |
| Hap_2 | GN6 | 0 | 2 | 0 | 1 | 0 | 0 | 0 | 3 | 0 | 3 | 0 | 3 | 0 | 3 | | 1 | 2 | | 0 | | 0 | | 1 | | 1 | | 0 | | 1 | | 1 | | 1 | | 0 | | 0 | | 0 | | 1 | | 1 |
| Hap_2 | GN24 | 0 | 2 | 0 | 1 | 0 | 0 | 0 | 3 | 0 | 3 | 0 | 3 | 0 | 3 | | 1 | 2 | | 0 | | 0 | | 1 | | 1 | | 0 | | 0 | | 1 | | 1 | | 0 | | 1 | | 0 | | 1 | | 1 |
| Hap_2 | F42 | 0 | 2 | 0 | 1 | 0 | 0 | 0 | 3 | 0 | 1 | 0 | 3 | 0 | 3 | | 1 | 2 | | 0 | | 0 | | 1 | | 1 | | 0 | | 0 | | 0 | | 1 | | 0 | | 0 | | 0 | | 1 | | 1 |
| Hap_2 | F46 | 1 | 5 | 0 | 1 | 0 | 0 | 0 | 4 | 0 | 0 | 1 | 3 | 0 | 3 | | 1 | 2 | | 0 | | 0 | | 0 | | 1 | | 1 | | 0 | | 0 | | 1 | | 0 | | 1 | | 1 | | 1 | | 3 |
| Hap_2 | F64 | 0 | 2 | 0 | 1 | 0 | 0 | 0 | 3 | 0 | 1 | 0 | 3 | 0 | 3 | | 1 | 2 | | 1 | | 0 | | 1 | | 1 | | 0 | | 1 | | 1 | | 1 | | 1 | | 1 | | 0 | | 1 | | 1 |
| Hap_2 | F45 | 0 | 1 | 0 | 1 | 0 | 0 | 0 | 3 | 0 | 1 | 0 | 3 | 0 | 3 | | 1 | 2 | | 0 | | 0 | | 1 | | 0 | | 0 | | 0 | | 1 | | 0 | | 0 | | 0 | | 0 | | 1 | | 1 |
| Hap_2 | F50 | 1 | 0 | 0 | 1 | 0 | 0 | 0 | 3 | 0 | 1 | 2 | 3 | 0 | 3 | | 1 | 2 | | 0 | | 0 | | 1 | | 0 | | 0 | | 0 | | 0 | | 0 | | 0 | | 1 | | 0 | | 1 | | 1 |
| Hap_2 | F62 | 0 | 0 | 0 | 1 | 0 | 0 | 0 | 3 | 0 | 0 | 1 | 3 | 0 | 3 | | 1 | 2 | | 1 | | 1 | | 1 | | 1 | | 0 | | 1 | | 1 | | 1 | | 1 | | 0 | | 0 | | 1 | | 1 |
| Hap_2 | F59 | 0 | 4 | 0 | 1 | 0 | 0 | 0 | 3 | 0 | 0 | 1 | 3 | 0 | 3 | | 1 | 2 | | 0 | | 0 | | 1 | | 0 | | 1 | | 0 | | 1 | | 0 | | 0 | | 1 | | 1 | | 1 | | 3 |
| Hap_2 | F61 | 0 | 0 | 0 | 1 | 0 | 0 | 0 | 3 | 0 | 0 | 1 | 3 | 0 | 3 | | 1 | 2 | | 1 | | 1 | | 1 | | 1 | | 0 | | 1 | | 1 | | 1 | | 1 | | 0 | | 0 | | 1 | | 1 |
| Hap_2 | XP65 | 0 | 5 | 0 | 1 | 0 | 0 | 0 | 4 | 0 | 0 | 1 | 3 | 0 | 3 | | 1 | 2 | | 1 | | 0 | | 1 | | 1 | | 1 | | 0 | | 1 | | 0 | | 0 | | 1 | | 1 | | 1 | | 3 |
| Hap_2 | XP56 | 1 | 1 | 0 | 1 | 0 | 0 | 0 | 3 | 0 | 2 | 0 | 3 | 0 | 3 | | 1 | 2 | | 0 | | 0 | | 0 | | 0 | | 0 | | 1 | | 1 | | 1 | | 0 | | 0 | | 0 | | 1 | | 1 |
| Hap_2 | XP136 | 0 | 5 | 0 | 1 | 0 | 0 | 0 | 4 | 0 | 0 | 1 | 3 | 0 | 3 | | 1 | 2 | | 1 | | 1 | | 1 | | 1 | | 1 | | 1 | | 0 | | 0 | | 0 | | 1 | | 1 | | 1 | | 3 |
| Hap_2 | XP20 | 1 | 2 | 1 | 1 | 0 | 0 | 0 | 3 | 0 | 4 | 0 | 3 | 0 | 3 | | 1 | 2 | | 1 | | 1 | | 1 | | 1 | | 1 | | 1 | | 1 | | 1 | | 1 | | 1 | | 0 | | 1 | | 1 |
| Hap_2 | XP34 | 3 | 5 | 0 | 1 | 0 | 0 | 0 | 3 | 0 | 0 | 1 | 3 | 0 | 3 | | 1 | 2 | | 1 | | 0 | | 1 | | 1 | | 0 | | 1 | | 0 | | 0 | | 0 | | 1 | | 1 | | 1 | | 3 |
| Hap_2 | XP51 | 4 | 5 | 1 | 1 | 0 | 0 | 0 | 3 | 0 | 0 | 1 | 3 | 0 | 3 | | 1 | 2 | | 1 | | 1 | | 1 | | 1 | | 1 | | 0 | | 1 | | 1 | | 0 | | 1 | | 1 | | 1 | | 3 |
| Hap_2 | XP68 | 0 | 0 | 0 | 1 | 0 | 0 | 0 | 3 | 0 | 0 | 1 | 3 | 0 | 3 | | 1 | 2 | | 1 | | 1 | | 1 | | 1 | | 0 | | 1 | | 1 | | 1 | | 1 | | 0 | | 0 | | 1 | | 1 |
| Hap_2 | XP78 | 1 | 0 | 0 | 1 | 0 | 0 | 0 | 3 | 0 | 3 | 0 | 3 | 0 | 3 | | 1 | 2 | | 0 | | 0 | | 1 | | 1 | | 0 | | 0 | | 0 | | 1 | | 0 | | 0 | | 0 | | 1 | | 1 |
| Hap_2 | XP140 | 0 | 0 | 0 | 1 | 0 | 0 | 0 | 3 | 0 | 1 | 0 | 3 | 0 | 3 | | 1 | 2 | | 0 | | 0 | | 1 | | 0 | | 0 | | 1 | | 1 | | 1 | | 0 | | 0 | | 0 | | 1 | | 1 |
| Hap_2 | XP31 | 1 | 2 | 1 | 1 | 0 | 0 | 0 | 3 | 0 | 3 | 0 | 3 | 0 | 3 | | 1 | 2 | | 0 | | 0 | | 1 | | 1 | | 0 | | 1 | | 1 | | 1 | | 0 | | 1 | | 0 | | 1 | | 1 |
| Hap_2 | XP60 | 0 | 6 | 0 | 1 | 0 | 0 | 0 | 4 | 0 | 0 | 1 | 3 | 0 | 3 | | 1 | 2 | | 0 | | 0 | | 1 | | 0 | | 1 | | 0 | | 1 | | 0 | | 0 | | 1 | | 1 | | 1 | | 3 |
| Hap_2 | XP61 | 0 | 5 | 0 | 1 | 0 | 0 | 0 | 3 | 0 | 0 | 1 | 3 | 0 | 3 | | 1 | 2 | | 1 | | 1 | | 1 | | 1 | | 1 | | 1 | | 0 | | 0 | | 0 | | 1 | | 1 | | 1 | | 3 |
| Hap_2 | XP7 | 0 | 5 | 0 | 1 | 0 | 0 | 0 | 4 | 0 | 0 | 1 | 3 | 0 | 3 | | 1 | 2 | | 1 | | 1 | | 1 | | 1 | | 1 | | 0 | | 1 | | 1 | | 1 | | 1 | | 1 | | 1 | | 3 |
| Hap_2 | XP90 | 3 | 5 | 0 | 1 | 0 | 0 | 0 | 3 | 0 | 0 | 1 | 3 | 0 | 3 | | 1 | 2 | | 1 | | 1 | | 1 | | 1 | | 1 | | 0 | | 1 | | 1 | | 1 | | 1 | | 1 | | 1 | | 1 |
| Hap_2 | GZ65 | 0 | 5 | 0 | 1 | 0 | 0 | 0 | 3 | 0 | 0 | 1 | 3 | 0 | 3 | | 1 | 2 | | 1 | | 0 | | 0 | | 0 | | 0 | | 0 | | 0 | | 0 | | 0 | | 0 | | 1 | | 1 | | 3 |
| Hap_2 | pd213169 | 1 | 0 | 0 | 1 | 0 | 0 | 0 | 3 | 0 | 1 | 2 | 3 | 0 | 3 | | 1 | 2 | | 0 | | 0 | | 1 | | 0 | | 0 | | 0 | | 0 | | 0 | | 0 | | 1 | | 0 | | 1 | | 1 |
| Hap_2 | GN21 | 2 | 5 | 0 | 1 | 0 | 0 | 0 | 4 | 0 | 0 | 1 | 3 | 0 | 3 | | 1 | 2 | | 1 | | 0 | | 1 | | 1 | | 1 | | 0 | | 1 | | 0 | | 0 | | 1 | | 1 | | 1 | | 3 |
| Hap_2 | GZ45 | 0 | 2 | 0 | 1 | 0 | 0 | 0 | 3 | 0 | 3 | 0 | 3 | 0 | 3 | | 1 | 2 | | 1 | | 1 | | 1 | | 1 | | 1 | | 1 | | 1 | | 1 | | 0 | | 1 | | 0 | | 1 | | 1 |
| Hap_2 | GZ94 | 4 | 5 | 1 | 1 | 0 | 0 | 0 | 3 | 0 | 0 | 1 | 3 | 0 | 3 | | 1 | 2 | | 0 | | 0 | | 0 | | 0 | | 0 | | 0 | | 1 | | 0 | | 0 | | 0 | | 1 | | 1 | | 3 |
| Hap_2 | GZ83 | 1 | 2 | 0 | 1 | 0 | 0 | 0 | 3 | 0 | 1 | 0 | 3 | 0 | 3 | | 1 | 2 | | 1 | | 1 | | 1 | | 1 | | 0 | | 0 | | 1 | | 1 | | 0 | | 1 | | 0 | | 1 | | 3 |
| Hap_2 | GZ78 | 2 | 5 | 0 | 1 | 0 | 0 | 0 | 4 | 0 | 0 | 1 | 3 | 0 | 3 | | 1 | 2 | | 1 | | 1 | | 0 | | 0 | | 1 | | 0 | | 0 | | 0 | | 0 | | 0 | | 1 | | 1 | | 3 |
| Hap_2 | YN56 | 0 | 5 | 0 | 1 | 0 | 0 | 0 | 4 | 0 | 0 | 1 | 3 | 0 | 3 | | 1 | 2 | | 1 | | 0 | | 1 | | 1 | | 1 | | 0 | | 1 | | 0 | | 0 | | 1 | | 1 | | 1 | | 3 |
| Hap_2 | pd11230 | 0 | 2 | 0 | 1 | 0 | 0 | 0 | 3 | 0 | 3 | 0 | 3 | 0 | 3 | | 1 | 2 | | 0 | | 0 | | 1 | | 1 | | 0 | | 0 | | 0 | | 1 | | 0 | | 0 | | 0 | | 1 | | 1 |
| Hap_2 | pd11221 | 1 | 2 | 0 | 1 | 0 | 0 | 0 | 3 | 0 | 1 | 0 | 3 | 0 | 3 | | 1 | 2 | | 0 | | 0 | | 1 | | 1 | | 0 | | 0 | | 1 | | 0 | | 0 | | 0 | | 0 | | 1 | | 1 |
| Hap_2 | pd11220 | 1 | 5 | 0 | 1 | 0 | 0 | 0 | 3 | 0 | 0 | 1 | 3 | 0 | 3 | | 1 | 2 | | 1 | | 0 | | 1 | | 1 | | 1 | | 0 | | 1 | | 0 | | 0 | | 1 | | 1 | | 1 | | 3 |
| Hap_2 | GN7 | 1 | 0 | 0 | 1 | 0 | 0 | 0 | 3 | 0 | 1 | 2 | 3 | 0 | 3 | | 1 | 2 | | 0 | | 0 | | 1 | | 0 | | 0 | | 0 | | 0 | | 0 | | 0 | | 1 | | 0 | | 1 | | 1 |
| Hap_2 | GN8 | 2 | 5 | 1 | 1 | 0 | 0 | 0 | 3 | 0 | 0 | 1 | 3 | 0 | 3 | | 1 | 2 | | 0 | | 0 | | 1 | | 1 | | 1 | | 0 | | 0 | | 1 | | 1 | | 1 | | 1 | | 1 | | 3 |
| Hap_2 | GN10 | 1 | 2 | 1 | 1 | 0 | 0 | 0 | 3 | 0 | 1 | 0 | 3 | 0 | 3 | | 1 | 2 | | 0 | | 0 | | 1 | | 1 | | 0 | | 0 | | 1 | | 0 | | 0 | | 0 | | 0 | | 1 | | 1 |
| Hap_2 | GN13 | 1 | 2 | 0 | 1 | 0 | 0 | 0 | 3 | 0 | 1 | 0 | 3 | 0 | 3 | | 1 | 2 | | 1 | | 1 | | 1 | | 1 | | 0 | | 1 | | 1 | | 1 | | 0 | | 1 | | 0 | | 1 | | 1 |
| Hap_2 | GN29 | 0 | 5 | 0 | 1 | 0 | 0 | 0 | 4 | 0 | 1 | 1 | 3 | 0 | 3 | | 1 | 2 | | 0 | | 0 | | 0 | | 0 | | 0 | | 0 | | 0 | | 1 | | 0 | | 1 | | 1 | | 1 | | 3 |
| Hap_2 | GN3 | 3 | 5 | 0 | 1 | 0 | 0 | 0 | 3 | 0 | 0 | 1 | 3 | 0 | 3 | | 1 | 2 | | 1 | | 0 | | 0 | | 0 | | 0 | | 0 | | 0 | | 0 | | 0 | | 0 | | 1 | | 1 | | 3 |
| Hap_2 | GZ116 | 0 | 5 | 0 | 1 | 0 | 0 | 0 | 4 | 0 | 1 | 1 | 3 | 0 | 3 | | 1 | 2 | | 0 | | 0 | | 0 | | 0 | | 0 | | 0 | | 0 | | 1 | | 0 | | 1 | | 1 | | 1 | | 3 |
| Hap_2 | GZ84 | 4 | 5 | 1 | 1 | 0 | 0 | 0 | 3 | 0 | 0 | 1 | 3 | 0 | 3 | | 1 | 2 | | 0 | | 0 | | 1 | | 1 | | 1 | | 0 | | 1 | | 1 | | 1 | | 1 | | 1 | | 1 | | 3 |
| Hap_2 | F107 | 0 | 1 | 0 | 1 | 0 | 0 | 0 | 3 | 0 | 1 | 0 | 3 | 0 | 3 | | 1 | 2 | | 0 | | 0 | | 1 | | 0 | | 0 | | 0 | | 1 | | 1 | | 0 | | 0 | | 0 | | 1 | | 1 |
| Hap_3 | XP33 | 0 | 2 | 1 | 1 | 0 | 0 | 0 | 3 | 0 | 1 | 1 | 4 | 0 | 3 | | 1 | 2 | | 0 | | 1 | | 0 | | 0 | | 0 | | 0 | | 0 | | 0 | | 0 | | 0 | | 0 | | 1 | | 1 |
| Hap_4 | XP10 | 0 | 2 | 1 | 1 | 0 | 1 | 0 | 3 | 0 | 1 | 1 | 4 | 0 | 3 | | 1 | 2 | | 0 | | 0 | | 0 | | 0 | | 0 | | 0 | | 0 | | 1 | | 0 | | 0 | | 0 | | 1 | | 1 |
| Hap_5 | XP81 | 0 | 2 | 1 | 1 | 0 | 1 | 0 | 3 | 0 | 1 | 1 | 4 | 0 | 3 | | 1 | 2 | | 0 | | 1 | | 1 | | 1 | | 0 | | 1 | | 1 | | 1 | | 0 | | 0 | | 0 | | 1 | | 1 |
| Hap_6 | PD13222 | 0 | 5 | 0 | 1 | 0 | 0 | 0 | 4 | 0 | 0 | 1 | 3 | 0 | 3 | | 1 | 2 | | 0 | | 0 | | 1 | | 0 | | 0 | | 0 | | 1 | | 1 | | 0 | | 0 | | 0 | | 1 | | 3 |
| Hap_7 | PD11229 | 3 | 2 | 1 | 1 | 0 | 0 | 0 | 4 | 0 | 1 | 1 | 3 | 0 | 3 | | 1 | 2 | | 0 | | 0 | | 1 | | 1 | | 1 | | 1 | | 1 | | 1 | | 0 | | 1 | | 0 | | 1 | | 1 |

* = Mating type; 1, 3 = self-fertile
